# Supplementary material for: The zinc-finger transcription factor Hindsight regulates ovulation competency of Drosophila follicles
Source: eLife. 2017 Dec 19;6:e29887. doi: 10.7554/eLife.29887 (PMC5768419; doi:10.7554/eLife.29887)
Supplement: Supplementary file 1. [file elife-29887-supp1.docx]

Supplementary file 1. The egg laying, egg distribution within the reproductive tract, and egg laying time of females with various genotypes

|  | Eggs laid in 2 days | | Egg distribution in 6hr | | | Egg laying time  (min) | | | |
| --- | --- | --- | --- | --- | --- | --- | --- | --- | --- |
| Genotype | n | Eggs laid/ female/day | n | Uterus with egg (%) | Oviduct with egg (%) | Total time | Ovulation time | Oviduct time | Uterus time |
| UAS-dcr2/+; 44E10-Gal4/(Ore-R) | 75 | 58.04 ± 1.15 | 59 | 44.07 ± 12.67 | 0.00 ± 0.00 | 22.74 ± 0.45 | 12.72 ± 2.89 | 0.00 ± 0.00 | 10.02 ± 2.89 |
| UAS-dcr2/Hnt^RNAi1^; 44E10-Gal4/+ | 75 | 37.73 ± 0.86*** | 73 | 26.03 ± 10.07 | 1.37 ± 2.67 | 34.99 ± .80 | 25.40 ± 3.63** | 0.48 ± 0.93 | 9.11 ± 3.53 |
| UAS-dcr2/Hnt^RNAi2^; 44E10-Gal4/+ | 75 | 31.34 ± 1.07*** | 83 | 44.58 ± 10.69 | 2.41 ± 3.30 | 42.12 ± .433 | 22.33 ± 4.59 | 1.01 ± 1.39 | 18.78 ± 4.55 |
| UAS-dcr2/+; 47A04-Gal4/(Ore-R) | 50 | 61.60 ± 2.02 | 57 | 35.09 ± 12.39 | 1.75 ± 3.41 | 21.43 ± 0.70 | 13.53 ± 2.72 | 0.38 ± 0.73 | 7.52 ± 2.67 |
| UAS-dcr2/Hnt^RNAi1^; 47A04-Gal4/+ | 50 | 31.03 ± 1.75*** | 54 | 25.93 ± 11.69 | 3.70 ± 5.04 | 42.54 ± 2.40 | 29.94 ± 5.45* | 1.58 ± 2.14 | 11.03 ± 5.01 |
| UAS-dcr2/Hnt^RNAi2^; 47A04-Gal4/+ | 35 | 41.22 ± 1.95*** | 96 | 45.83 ± 9.97 | 0.00 ± 0.00 | 32.02 ± 1.52 | 17.35 ± 3.30 | 0.00 ± 0.00 | 14.68 ± 3.27 |
| UAS-dcr2/+; 44E10-Gal4/(Ore-R) | 125 | 68.74 ± 1.08 | 61 | 45.90 ± 12.51 | 1.64 ± 3.19 | 19.20 ± 0.30 | 10.07 ± 2.41 | 0.31 ± 0.61 | 8.81 ± 2.41 |
| UAS-dcr2/Hnt^RNAi1^; 44E10-Gal4/+ | 100 | 38.14 ± 0.64*** | 59 | 20.34 ± 10.27 | 8.47 ± 7.11 | 34.61 ± 0.58 | 24.64 ± 4.02** | 2.93 ± 2.46 | 7.04 ± 3.56 |
| UAS-dcr2/Hnt^RNAi1^; 44E10-Gal4/UAS-RREB1::GFP | 55 | 20.63 ± 1.29*** | 46 | 13.04 ± 9.73 | 17.39 ± 10.95 | 63.99 ± 4.01 | 44.52 ± 8.95*** | 11.13 ± 7.04 | 8.35 ± 6.25 |
| UAS-dcr2/Hnt^RNAi2^; 44E10-Gal4/+ | 90 | 36.47 ± 0.76*** | 58 | 25.86 ± 11.27 | 0.00 ± 0.00 | 36.20 ± 0.76 | 26.84 ± 4.12*** | 0.00 ± 0.00 | 9.36 ± 4.08 |
| UAS-dcr2/Hnt^RNAi2^; 44E10-Gal4/UAS-RREB1::GFP | 100 | 56.66 ± 1.14** | 61 | 32.79 ± 11.78 | 9.84 ± 7.47 | 23.30 ± 0.47 | 13.37 ± 2.90 | 2.29 ± 1.74 | 7.64 ± 2.75 |
| UAS-dcr2/Kr^IF-1^; 44E10-Gal4/UAS-RREB1::GFP | 55 | 50.43 ± 2.77*** | 51 | 27.45 ± 12.25 | 1.96 ± 3.81 | 26.17 ± 1.44 | 18.47 ± 3.43 | 0.51 ± 1.00 | 7.18 ± 3.23 |

All data are plotted as average ± 95% C.I.

One day is considered as 22h in 29 °C.

* P<0.05, ** P<0.01, and *** P<0.001. One-way ANOVA with *post hoc* Fisher’s Least Significant Difference test is used for egg laying, and Z-score test is used for egg laying time assuming normal distribution.
